# Managing and preparing for eye emergencies

**Published:** 2018-11-09

**Authors:** John Buchan, Seema Verma

**Affiliations:** 1 Consultant Ophthalmologist: International Centre for Eye Health, London School of Hygiene and Tropical Medicine, London, UK.; 2 Consultant Ophthalmologist and President of the British Emergency Eye Care Society, London, UK.

**Eye emergencies can have devastating consequences. We should do everything in our power to ensure that patients' sight – and lives – can be saved.**

**Figure:**
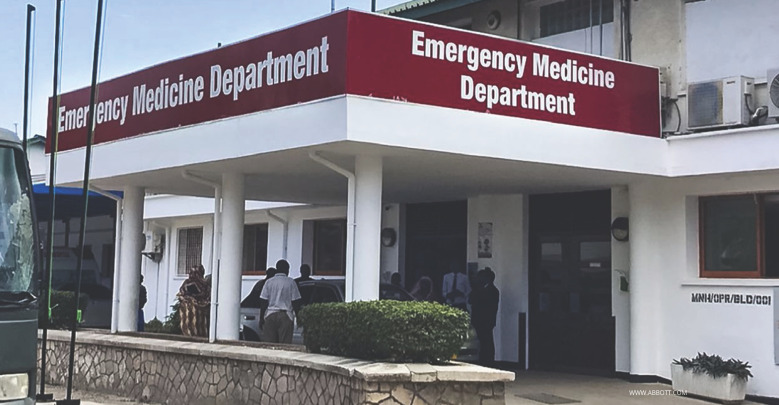
Early detection of eye emergencies is essential – whether in the community or in hospital. TANZANIA

Whilst most eye problems progress gradually and can be dealt with in routine clinical practice, there are times when action can save someone's sight, or even their life.

This issue of the *Community Eye Health Journal* looks at some of the conditions for which emergency intervention is essential and where delay, of even a few hours, can have devastating consequences.

Anyone working in eye care can learn to identify an eye emergency. Some emergencies, such as retinal detachment (p. 63), must be attended to by a specialist surgeon. In this case, the role of the front-line eye health worker is to identify patients who need urgent treatment and refer them. Other patients with acute conditions, such as compression of the optic nerve, must be treated immediately. In this case, front-line members of the eye care team should be prepared to perform a sight-saving lateral canthotomy (p. 62).

In each example, the key to saving sight is preparation, whether that is developing an effective referral pathway for retinal detachment, ensuring that access to acetazolamide for acute angle-closure glaucoma is possible outside of normal working hours (p. 64), or practising setting up for an anterior vitrectomy (p. 65). If we are not ready and waiting for these emergencies, we will miss the opportunity to prevent blindness or save someone's life.

